# Quality of Life Among Thai Workers in Textile Dyeing Factories

**DOI:** 10.5539/gjhs.v7n3p274

**Published:** 2014-11-30

**Authors:** Wirin Kittipichai, Rattanaporn Arsa, Ann Jirapongsuwan, Chatchawal Singhakant

**Affiliations:** 1 Faculty of Public Health, Mahidol University, Bangkok, Thailand

**Keywords:** quality of life, textile dyeing, work environment, safety at work, Thai worker

## Abstract

The purpose of a cross-sectional study was to investigate factors influencing the quality of life among Thai workers in textile dyeing factories. Samples included 205 Thai workers from five textile dyeing factories located in the suburban area of Bangkok in Thailand. Data were collected with a self-administered questionnaire. Scales of the questionnaire had reliability coefficients ranging from 0.70–0.91. The results revealed that the overall quality of life among workers was most likely between good and moderate levels, and the percentage-mean score was 74.77. The seven factors associated with the overall quality of life were co-worker relationships, safety at work in the dimension of accident prevention, job characteristics, supervisory relationships, welfares, marital status, and physical environment. Furthermore, co-worker relationships, accident prevention, and marital status were three considerable predictors accounted for 23% of the variance in the overall quality of life among workers in textile dyeing factories.

## 1. Introduction

The economic growth of Thailand has been increasing rapidly especially from the industrial production and exportation. Numbers of labors have been moving from an agricultural sector to the industrial sector. One of the key success factors of an organization is “humans”. It is necessary for the organization to retain good employees (i.e. those who had shown quality performance) to stay with a long term commitment with the organization. However, due to the growth of the industrial sector, most organizations tended to focus on increasing their productions. They employed more people, increased the working hours. As well as, at the same time their employees had to do their work correctly and precisely, which might result in fatigue and occupational stress (Mueanthap & Mapudh, 2008). During 2007-2011, the incidents of occupational accidents or illnesses were reported as follows: a cut/stick injury by an object (23.22%), a hit/crash by an object (16.31%), and an eye irritation by a chemical substance (15.91%) (Compensation fund, 2012). Such an accident might be contributed by a long period of working hard among the workers, which could lead to physical fatigue, carelessness, low level of immunity, and poor mental health. Ultimately, this would result in “work-life imbalance”; and the employees would lose their chance to enjoy a good quality of life.

“Quality of Life” is the perceived satisfaction of individuals about living their life in society. This perception has a relation with a goal and expectation within the contexts of culture, values, norms, and other related issues (WHO, 1997). Additionally, there have a relation with the type of their lifestyle; that is consistent with their basic needs such as having a good wellbeing, a good health, an employment, and an ability to be a good citizen (Kittipichai, Wongpinpetch, Niramitchainont, & Fongloa, 2012). In other word, if the individuals can do anything that they are satisfied with, and live in a good environment; then they would have a good quality of life. This thought is in line with the concept of “Happy Workplace” that emphasizes the “work-life balance” among the workers, i.e. the balance between their personal life, family, and society. This concept focuses the importance on Happy 8: happy body, happy relax, happy heart, happy soul, happy family, happy society, happy brain, and happy money (Thai Health Promotion Foundation, 2009). All of these serve as the “indicators for assessing a good quality of life among the workers in the organizations”.

As “humans” serve as a key success factor of an organization; therefore, it is necessary that the quality of life must be ensured for all of the workers in the organization. Consequently, the quality of the workers would reflect the quality of the organization. The findings of previous studies showed that there were both positive and negative factors that had affected the quality of life among workers in the industrial sector. The examples of positive factors included the status of a full-time employment (Chutipongnawin, 2006; Singchongchai, 1996), satisfaction with working situation, job satisfaction and the pride about the job (Jingjitra, 2011), relationship with co-workers and supervisor, and work environment (Damrongsak & Harnirattisai, 2012; Nuntaboot, 2012; Phewkleang & Rewmongkhol, 2013). The negative factors were such as the type of work in which the workers had to be exposed to a chemical substance (Chutipongnawin, 2006), and working hours (Singchongchai, 1996). Therefore, there were several levels of factors that had affected the quality of life. Concerning the “Ecological Model of Health Behavior”; factors could be divided into three levels: individual level (i.e. age, sex, education, attitude, beliefs, employment status, and the personality of individuals); intrapersonal level (i.e. the interaction with family, close friends, co-workers, and co-members of a same organization; and organizational level (i.e. organizational environment, rules, regulations, laws, and policy) (Glanz, Rimer, & Lewis, 2002).

In Thailand, the textile dyeing factory is an important component of the national textile industrial system, as well as the national economy. Most production processes in the dyeing factories were chemical processes in which a lot of chemicals and dyes were used. So, the workers in the production lines exposed to hazardous chemicals almost all the time resulting in the negative effects to their health. These hazards could occur in an acute or a chronic pattern depending on the situation or the environment of the unsafe workplace. Apart from chemical hazards, the workers in textile dyeing factories also had a chance for an exposure to other hazards such as physical factors, which included illumination, sound, vibration and heat; ergonomic factors, which were related to working posture; and psychosocial factors, such as interpersonal relationships, and job characteristics. According to the accident and incident investigation report for industrial factories in 2012, there were collectively three incidents of the fire at the textile dyeing factories in Bangkok’s neighboring provinces. (Department of Industrial works, 2012). Therefore, it is evident that the workers in textile dyeing factories are at a high risk of an exposure to various hazards caused by several factors. As well, if the factories’ administration does not have any preventive measures in place, or if the workers become careless and ignore the practice of the preventive measures. The workers may be affected by the occupational hazards which would create a further effect to their quality of life.

This research aimed to explore the quality of life among Thai workers in textile dyeing factories and related factors. The factors supposed to cause an effect on the life quality of the workers were divided into three groups: Factors at individual level (i.e. sex, marital status, employment status, and tenure of work); Factors at interpersonal level (i.e. supervisory relationships and co-worker relationships); and Factors at organizational level (i.e. job characteristics, welfares, safety at work, physical environment, chemical environment, and ergonomic environment). Findings of this study would guide the policy maker of the textile dyeing factories to take into account to the promotion of quality of life for their workers. To facilitate the workers would be a quality human and become valuable for driving the organization towards a quality organization and a happy workplace.

## 2. Methods

### 2.1 Research Design and Sample

This research was a cross-sectional survey study conducted among 216 Thai workers who worked in the production lines and the color testing laboratories from five textile dyeing factories. Factories are located in the neighboring provinces known as Bangkok Metropolitan Region (BMR) which covered five provinces: Samutprakarn, Pathumthani, Samutsakorn, Nakornpatom and Nonthaburi. Workers who were willing to participate in the study were asked to sign a consent form for participation in research. A random sampling method was used. Data was collected between March and May 2014. This study was approved by the ethics committee for research in human subjects of the faculty of Public health, Mahidol University (Ref. No. MUPH 2014-086).

### 2.2 Research Instruments

The research instrument was a self-administered questionnaire comprising four parts. *Part 1:* Demographics data (including sex, age, income, education, marital status, tenure of work, employment status, and department) comprised 11 items (with the multiple-choice answers for the respondent to choose from; and the blanks for them to fill in based on the truth). *Part 2:* The factors at interpersonal level included five items about supervisory relationships and five items about co-worker relationships, with a 5-point rating scale from the mostly (5) to rarely (1). *Part 3:* The factors at organizational level comprised 42 items about job characteristics (3 items), the welfares (4 items), safety at work in the dimension of an accident prevention (8 items), physical environment (10 items), chemical environment (6 items) and the hazard perception in non-ergonomic ally working posture as an ergonomic environment (6 items) used the scales of 5-point rating scale from the mostly (5) to rarely (1). This part also consisted of another 5 items with two choices of answer provided: Yes (1) and No (0), there were about safety at work in the dimension of the protective personal equipment (PPE) in workplace. *Part 4:* The Quality of Life (QOL) questions were adapted from the Happinometer (Kittisuksathit, Tangchonlatip, Jaratsit, et al., 2012) and consisted of 34 items. Of these, six items were either those provided with multiple-choice answers or those prepared with the blanks for the respondents to fill in; while the other 28 items prepared as a 5-point rating scale from the mostly (5) to rarely (1). Levels of QOL, score of QOL scale were to be categorized by percentage-score cut-off point: good (75–100), moderate (50–74.99), and poor (0–49.99). Content validity was reviewed and approved by three experts in environmental science, occupational health science, and behavioral science. The internal consistency reliability coefficients were found between 0.70 and 0.91.

### 2.3 Statistical Analysis

The SPSS for Windows was used for data analysis. The statistics used included descriptive statistics (i.e. frequency, percentage, mean, median, and standard deviation) and inferential statistics (i.e. Pearson’s product-moment correlation coefficient, and Stepwise Multiple Regression Analysis). The statistical significance was set at the level of less than .05.

## 3. Results

A total of 205 questionnaires from Thai workers in textile dyeing factories were completed. Worker respondents worked in the dyeing department (47.2%), the finishing/quality testing department (24.1%), the color testing department (16.7%), the fabric preparation department (8.3%) and the packaging department (3.7%). The numbers of male and female were almost equal. The average age of them was 34.33±9.89 years old, and almost 60% of them aged between 26-45 years. More than half of them held the marital status as being married/couple, and mostly all of them finished high school. Their incomes ranged from 6,000-50,000 Baht per month (averaged 10,500 Baht/month). Nearly two-thirds of the samples held employment status as a full time. The tenure of work ranged from 1-36 years (averaged 11 years), and almost six-tenths of participants worked overtime (Table 1).

**Table 1:** Information of participants

Items	Frequency	Percentage
**Sex**		
Female	106	51.7
Male	99	48.3
**Age** (years old)		
18–25	56	27.3
26–35	60	29.3
36–45	57	27.8
46–60	32	15.6
Range = 18–60, Mean±S.D = 34.33±9.89		
**Marital status**		
Married/Couple	105	51.2
Single	87	42.4
Widowed/Divorced/Separated	13	4.4
**Education**		
Primary school	20	9.7
High school	94	45.9
Vocational certificate or Diploma	45	21.9
Bachelor or more	46	22.4
**Income** (Baht per month)		
<8,000	55	26.8
8,000–10,000	43	21.0
10,001–15,000	54	26.3
>15,000	53	25.9
Range=6,000–50,000 Median=10,500, Q1=8,000, Q3=17,000		
**Employment status**		
Full time	127	62.0
Part time/temporary	78	38.0
**Tenure of work** (years)		
<1	42	20.5
1–5	54	26.3
6–10	28	13.7
11–15	33	16.1
16–36	48	23.4
Range=1–36, Mean±S.D.=10.78±9.24		
**Work overtime**		
Yes	121	59.0
No	84	41.0
**Work injury**		
Yes	48	23.4
No	157	76.6

The assessment of Thai workers’ life quality showed that the percentage score of the overall QOL ranged from 52.6-94.0, and percentage-mean score was 74.77. About half of respondents (50.9%) had a moderate level while the other half (49.1%) had a good level of QOL. Considering each dimension of QOL, it was found that the soul dimension had the highest percentage-mean score followed by heart, brain, society, family, relax, body, and money, respectively (Table 2).

**Table 2:** Score and Level of Quality of life

Quality of life	n of item	Raw score	Percentage score		Level of QOL (%)		
		
		Range	Range	Mean±S.D.	Good	Moderate	Fair
Overall QOL Dimension:			52.6–94.0	74.77±7.54	49.1	50.9	0.0
- The soul	5	13–25	52.0–100.0	81.73±10.89	78.0	22.0	0.0
- The heart	4	10–20	50.0–100.0	79.60±12.70	68.1	31.9	0.0
- The brain	3	5–15	33.3–100.0	76.97±13.99	52.3	43.5	4.2
- The society	5	11–25	44.0–100.0	75.62±9.97	58.4	40.7	0.9
- The family	3	3–15	20.0–100.0	73.86±19.03	46.3	41.1	12.6
- The relax	3	6–15	40.0–100.0	71.01±11.90	33.5	61.4	5.1
- The body	7	14–32	40.0–91.0	69.46±10.90	28.9	65.3	5.8
- The money	4	7–20	35.0–100.0	67.36±12.73	33.0	60.8	6.2

Reliability coefficients of QOL scale = 0.79

The percentage-mean score of supervisory relationships was higher than that of the co-worker relationships. With regards to the perception about work environment and safety at work, the perception of PPE in the workplace was the highest percentage-mean score, followed by the perception about job characteristics, accident prevention, physical environment, chemical environment, welfares, and ergonomic environment, respectively (Table 3).

**Table 3:** Characteristics of scales

Scales	Reliability coefficients	Raw score		Percentage score	
	
Range	Range	Mean±S.D.
Supervisory relationships	0.87	11 – 25	44.0 – 100.0	81.49±13.68
Co-worker relationships	0.88	12 – 25	48.0 – 100.0	79.52±12.99
Welfares	0.83	6 – 20	30.0 – 100.0	65.93±14.64
Job characteristics	0.70	9 – 15	60.0 – 100.0	82.41±11.66
Safety at work				
- PPE in the workplace	0.83	0 – 5	0.0 – 100.0	88.53±21.98
- Accident prevention	0.80	15 – 40	38.0 – 100.0	82.28±12.51
Physical environment	0.86	17 – 60	30.0 – 100.0	66.89±15.03
Chemical environment	0.91	6 – 30	20.0 – 100.0	66.35±15.03
Ergonomic environment	0.91	6 – 30	20.0 – 100.0	60.93±22.34

Pearson’s product moment correlation coefficient analysis was employed for finding the association between factors and the overall QOL among workers in textile dyeing factories. Factors that significantly correlated with the overall QOL among workers were co-worker relationships, accident prevention, job characteristics, supervisory relationships, welfares, (r=0.36, 0.36, 0.33, 0.28, 0.24, p<.001), marital status (r=0.18, p<.01), and physical environment (r=0.16, p<.05), while coefficients between sex, employment status, tenure of work, PPE in the workplace, chemical environment and ergonomic environment and overall QOL were not significant (p>.05). However, the physical and chemical environments were found in high correlation with an ergonomic environment (r=0.67, 0.73, p<.01) (Table 4).

**Table 4:** Pearson’s product-moment correlation coefficients

Factors	QOL	1	2	3	4	5	6	7	8	9	10	11	12
Sex^a^ (1)	.05												
Marital status^b^ (2)	.18**	-.08											
Employment status^c^ (3)	.04	.26***	-.01										
Work tenure (4)	.09	.01	.39***	.13									
Supervisory rel (5)	.28***	.12	.04	.07	-.06								
Co-workers rel (6)	.36***	.06	-.07	.06	-.17*	.48***							
Job characteristic (7)	.33***	.15*	.05	.05	.03	.38***	.44***						
Welfares (8)	.24***	-.03	-.04	-.14*	.02	.22**	.25***	.11					
PPE in the workplace (9)	.12	.01	.02	-.04	-.01	.23**	.13	.09	.07				
Accident prev (10)	.36***	.06	.17	.16*	.21**	.29***	.22**	.42**	.24***	.13			
Physical envi.(11)	.16*	.21**	.06	.30***	.15*	.33***	.19***	.16*	.08	.13	.24**		
Chemical envi (12)	.08	.18**	.85	.29***	.22**	.20**	.08	.12	.03	.01	.20**	.09*	
Ergonomic envi	.06	.12	.05	.27***	.12	.12	.04	.13	.07	.05	.13	.67***	.73***

Reference group: sex^a^: male, marital status^b^: = non-married, employment status: part time.

* p-value <0.05,

** p-value < 0.01,

*** p-value < 0.001.

The seven factors were significantly associated with overall QOL. These variables were analyzed to see how it influences overall QOL by using Stepwise MRA. The inter-correlation coefficients among predictors were used to verify the multicollinearity problem. The finding showed that inter-correlation coefficients were not highly correlated (r<0.7) (Table 4). In addition, the collinearity statistic showed that the tolerance value closed up zero, and VIF valueless than 10. Likewise, the result of Stepwise MRA revealed that the three considerable predictors of overall QOL among workers in textile dyeing factories were the co-worker relationships (β=0.31), safety at work in the dimension of accident prevention (β=0.26), and marital status (β=0.16), which accounted for 24% of the variance in the overall QOL (Table 5). This relationship might be shown in and the equation as:

**Table 5:** Stepwise multiple regression analysis of the overall quality of life

Variables	B	Beta	t-value	Tolerance	VIF
Co-worker relationships	0.63	0.32	5.13***	0.94	1.06
Accident prevention	0.34	0.26	4.14***	0.92	1.09
Marital status (Married)	2.02	0.16	2.52**	0.96	1.04
Constant	49.9		15.46***		

R^2^=0.24, R^2^ adjusted=0.23, Durbin-Watson=2.01.


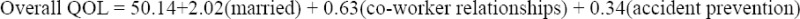


## 4. Discussion

The findings of this study have shown that, for the overall QOL among Thai workers in textile dyeing factories, the moderate and good levels of the QOL were found in a similar percentage. This finding is similar to the study of Somboonlertsiri (2013) which found that the score of overall QOL among the operational workers in the IRPC Public Company limited, producer of integrated petrochemical products, in Thailand had a high level. In this study, the quality of life among Thai workers was assessed in eight dimensions. Based on the findings, the different dimensions of the QOL could be classified into 2 groups: (1) the group in which more than one-half of participants had a good level in the quality of life, which comprised four dimensions, namely, the soul, the heart, the brain, and the society; and (2) the group in which more than fifty percent of the samples had a moderate level, which comprised four dimensions included the money, the body, the relax, and the family. This survey was the assessment quality of life in relation with facets of their works and families.

The findings also showed that the lowest percentage-mean score of the QOL in the money dimension. It was evident that the Thai workers in their working-age had the burdens in taking responsibility for themselves and their families. Thus, it might be possible for them to have been engaged in some loans and debts. Also, their incomes might not be sufficient to cover their expenses due to the lack of job security (about four-tenths of them had been facing this situation) which resulted in unsteady amount of wages that they could earn in each month. Another reason was that they had just started their career, and were in the first five years of working; so their savings capacity was low. They had to work overtime or work harder to gain more income and that took away the time that they could spend with their family. As they could spare only a minimal amount of time for family, the happiness in family would also be minimal, and the time for taking a rest and for relaxation would be minimal as well. This situation affected physical and mental health, caused physical fatigue and weakness, and the persons would develop stress, would have a lower level of immunity and would be more susceptible to illnesses. However, as people in Thai society spiritually had generosity towards each other, cared for, had thoughtfulness about, and paid attention to people around them. For that reason, the workers might be able to live their life in society without much stress. Also, due to the development and advancement of technologies, they had wanted to develop themselves in order to increase their skill and capability, as well as the progress in life. By developing themselves as such, the QOL in the dimension of psychosocial issues and the eagerness to learn of most workers were in a good level. Meanwhile, the QOL in the dimension of work and family among most workers was in a moderate or poor level. Therefore, in order to ensure a good quality of life, work-life balance is required.

The findings showed that supervisory relationships, co-worker relationships, safety at work in the dimension of accident prevention, marital status, job characteristics, welfares, and physical environment were significantly correlated with the overall QOL. That is; co-worker relationships, the safety at work in accident prevention, and the marital status were able to work together in predicting the overall QOL among Thai workers in textile dyeing factories. This result could be explained as follows. About the factors at individual level, the persons hold the marital status as being married or couple. They would have someone that would care for them, encourage them, and serve as a counterpart in finding a solution for problems. Like living with a spouse, the persons would feel relaxed and would be able to receive care in several aspects, physically and mentally. These results are according to a study conducted in some informal workers in Thailand (Kanthvanna, 2007). Meanwhile, in this study, sex and tenure of work were not correlated with the overall QOL. This conclusion is consistent with previous studies in Thailand; a study conducted in some of the industrial factory workers (Kwangkeaw, 2004). And another one conducted in some workers in the frozen seafood business (Jingjitra, 2011). About the factors at the interpersonal level, co-worker relationships were an important one; given that co-workers were very important, and they had to work together all the time. When a problem arose from work, they could request assistance or seek consultation from their co-workers. Politely talking with each other and honoring each other would lead to the happiness in doing the work and help reduce the stress from work. This finding is according to the study of Chang, Liu, Hwang, Chen and Lu (2010) in Taiwan. Concerning the supervisory relationships with, though it was correlated with the overall QOL, however, it could not serve as a key predictor. A possible explanation would be that the perceived relationship with supervisors among the workers might not be different from each other because they might share the same supervisor. Therefore, they would have similar perception and feelings about the supervisor.

About the factors at the organizational level concerning the environment in the factories as well as job characteristics and the welfares provided to the workers. The textile dyeing factories selected as the samples in this study were the ones that had been accredited with various standards of environmental management, such as ISO 9001:2000, ISO 14001:2004 and BSI-OHSAS 18001. Therefore, the availability of PPE for the workers was important and essential for the factories to ensure. The findings of this study showed that more than eight-tenths of the workers reported that they had been provided with PPE in the workplace. On the other hand, also based on the findings, workers still faced a high risk due to improper environment management. They had perceived risk of being endangered by ergonomic, chemical, and physical environments; percentage-mean scores of them were in the range of 60-67. An explanation based on the ecological model of health behavior (Glanz, Rimer, & Lewis, 2002) could be that when the persons were in the same environment. They would be affected by such environment, and that they would share a similar perception and behavior. The difference in the perception found in each level was contributed by a smaller level of factors. Such as the fact that, as the workers worked in a factory, the environment inside the factory would be considered as a factor at organizational level. The reason for the workers to have a different perception about the factors at organizational level might be that they also had different perceptions about the factors at the interpersonal level and about those at individual level. It could be highlighted that this study has successfully achieved one important finding.

That is, despite the fact that several factors at organizational level, including job characteristics, welfares, safety at work, physical environment, chemical environment, and ergonomic environment, were correlated with the overall QOL. This conclusion is according to previous studies conducted in some workers of the textile industries (Kowsalyadevi & Kumar, 2013; Valarmathi & Bhalakarishnan, 2013; Rathamani & Ramchandra, 2013; Li, Ray, Gao et al., 2006), and some Thai workers of the Siam Cement (Thung Song) Co., Ltd. (Pol-in, 2006). However, only the factor safety at work in the dimension of accident prevention could serve as a vital predictor of the overall QOL among Thai workers. This factor was related to the organization of the training on the use of PPE in the workplace and on the topic of safety at work. This made the workers feel confident in doing their work, feel safe, and feel relieved from any worries about various occupational risks.

However, though the factories had organized such training to provide knowledge and increase the skills of the workers in preventing the hazards in the workplace. Even so in reality, some workers had never gone through such training, or only small number of the workers had gone through it (in other word, the coverage of the training was not comprehensive). This preparation might be contributed by the difference in their employment status. That is about four-tenths of the samples were hired on a part-time or temporary basis. So, when they came to work during a period when no training took place; this specific group of workers would lack an awareness, and lack knowledge and skills for doing their work safely. They would, therefore, have a risk of an occupational injury. Study data also showed that more than one-fifth of participants used to experience an occupational injury. Caused by they were the carelessness in doing their work, and lack of the awareness about the need to wear PPE in the workplace. This information is according to previous studies conducted in the textile industries that found healthy working conditions has influenced the QOL among workers (Rathamani & Ramchandra, 2013; Valarmathi & Bhalakarishnan, 2013). In addition to unhygienic workplace environment was some of the risk factors to health problems among workers in tobacco industries (Elias & Saha, 2009). Therefore, the administrators of factories should be aware of this and place importance on ensuring. Every worker shall be covered with the knowledge and skill training sessions for the prevention of any possible occupational or environmental hazards inside the factories. This idea would create a direct impact on the quality of life among workers, and on the work quality of the organization.
